# Neuroprotective Effects of Guanosine in Ischemic Stroke—Small Steps towards Effective Therapy

**DOI:** 10.3390/ijms22136898

**Published:** 2021-06-27

**Authors:** Karol Chojnowski, Mikolaj Opielka, Wojciech Nazar, Przemyslaw Kowianski, Ryszard T. Smolenski

**Affiliations:** 1 Faculty of Medicine, Medical University of Gdańsk, Marii Skłodowskiej-Curie 3a, 80-210 Gdańsk, Poland; karole97@gumed.edu.pl (K.C.); wojciech.nazar@gumed.edu.pl (W.N.); 2 Department of Biochemistry, Medical University of Gdansk, 1 Debinki St., 80-211 Gdansk, Poland; 3 International Research Agenda 3P—Medicine Laboratory, Medical University of Gdańsk, 3A Sklodowskiej-Curie Street, 80-210 Gdansk, Poland; 4 Department of Anatomy and Neurobiology, Medical University of Gdansk, 1 Debinki Street, 80-211 Gdańsk, Poland; przemyslaw.kowianski@gumed.edu.pl; 5 Institute of Health Sciences, Pomeranian University of Słupsk, Bohaterów Westerplatte 64, 76-200 Słupsk, Poland

**Keywords:** guanosine, stroke, neuroprotection, neuroinflammation, purinergic signaling

## Abstract

Guanosine (Guo) is a nucleotide metabolite that acts as a potent neuromodulator with neurotrophic and regenerative properties in neurological disorders. Under brain ischemia or trauma, Guo is released to the extracellular milieu and its concentration substantially raises. In vitro studies on brain tissue slices or cell lines subjected to ischemic conditions demonstrated that Guo counteracts destructive events that occur during ischemic conditions, e.g., glutaminergic excitotoxicity, reactive oxygen and nitrogen species production. Moreover, Guo mitigates neuroinflammation and regulates post-translational processing. Guo asserts its neuroprotective effects via interplay with adenosine receptors, potassium channels, and excitatory amino acid transporters. Subsequently, guanosine activates several prosurvival molecular pathways including PI3K/Akt (PI3K) and MEK/ERK. Due to systemic degradation, the half-life of exogenous Guo is relatively low, thus creating difficulty regarding adequate exogenous Guo distribution. Nevertheless, in vivo studies performed on ischemic stroke rodent models provide promising results presenting a sustained decrease in infarct volume, improved neurological outcome, decrease in proinflammatory events, and stimulation of neuroregeneration through the release of neurotrophic factors. In this comprehensive review, we discuss molecular signaling related to Guo protection against brain ischemia. We present recent advances, limitations, and prospects in exogenous guanosine therapy in the context of ischemic stroke.

## 1. Introduction

Stroke is one of the top causes of death worldwide and the leading cause of permanent disability in developed countries, affecting approximately one in six people in their lifetime worldwide [1,2,3]. In 2019, on a global scale, ischemic stroke and hemorrhagic stroke accounted for 77.2 million (76%) and 29.1 million (24%) cases of stroke, respectively [4]. Ischemic stroke is characterized by a transient or permanent reduction of cerebral blood flow, causing the depletion of oxygen and glucose levels, uncontrolled depolarization, energy deficit, excitotoxicity, and tissue infarct, thus disturbing physiological cellular function [5]. The central nervous system (CNS) is specifically sensitive to ischemia; thus, a thrombolytic or mechanical restoration of blood flow in a narrow time window remains the treatment of choice for limiting postischemic brain injury [3]. The ischemic core is surrounded by a transition zone—an ischemic penumbra composed of both normal and functionally impaired cells. Ischemic tissue damage develops slower in the penumbra; therefore, recent studies prompted the creation of therapeutic protocols, which enable clinicians to use thrombolytic therapy and thrombectomy in selected patients during extended time windows. For instance, thrombectomy is advised up to 24 h in eligible patients who have a mismatch between clinical deficits and infarct volume [6,7,8]. However, clinical evidence demonstrates that in the majority of stroke patients, slow brain injury evolution is observed in hours-to-days time intervals, which may be caused by reperfusion injury and activation of immunoinflammatory mechanisms [9]. Therefore, a neuroprotective therapy protecting neurons in the penumbra against ischemic and reperfusion injury is still highly demanded [10]. Despite a plethora of proposed neuroprotective drugs, including antioxidants, neuropeptides, microRNAs, anti-inflammatory drugs, and antiexcitatory drugs, their status is far from being clinically well established; thus, the question about pleiotropic neuroprotectant(s) in stroke therapy remains unanswered.

In recent years, guanosine (Guo), a part of a guanine-based purinergic system, emerged as a novel neuroprotectant and neuromodulator in CNS disorders. In this comprehensive review, we describe in depth the role and effects of extracellular guanosine in in vitro and in vivo models of ischemic stroke. The main objective of our study was to introduce the concept of Guo as a new potential neuroprotectant and/or neurotrophic agent supplementing the currently available ischemic stroke treatment methods. Starting from the observations of endogenous guanosine release under ischemia, we explore the concept of the therapeutic potential of exogenous guanosine. We discuss the molecular targets in relation to the neuroprotective and neurotrophic effects of extracellular guanosine. We also present the future directions in approaches to guanosine application in ischemic stroke.

We performed a literature search of Medline, Scopus, and Embase databases published between 1 January 1990 and May 2021 to identify studies addressing the role of guanosine in ischemic stroke management in both in vitro and in vivo ischemic stroke models. To the best of our knowledge, there is currently no available experimental data regarding the neuroprotective effects of Guo in hemorrhagic stroke; thus, the scope of the study was limited to ischemic stroke models. Included studies comprised both original and review articles. Searches were independently conducted by two of the authors. We searched Medical Subject Headings (MeSH) terms limited to the English language in multiple combinations, including brain ischemia/ischemic stroke, oxygen glucose deprivation, guanosine, neuroprotective agent, and neuroprotection. Additionally, references from included studies were screened for relevant studies.

## 2. The Physiological Role and Signaling Targets of Endogenous Guanosine in Central Nervous System

### 2.1. Cellular Location, Release, and Metabolism of Guanine Derivatives in the Brain

Guanine derivatives (GDs) include guanosine 5′-triphosphate (GTP), guanosine 5′-diphosphate (GDP guanosine 5′-monophosphate (GMP), and guanosine. GTP and GDP have mostly been recognized as intracellular modulators of G-protein activity [11]. However, apart from the regulation of G proteins, GDs are also found to be involved in the extracellular signaling in the CNS [12]. Thus, analogously to the adenosine-based purinergic system, a signaling system based on GDs has been proposed [13]. 

GTP is co-stored in synaptic vesicles and released with ATP, suggesting the role of GTP in neurotransmission [14,15]. The pool of GDs in the brain is located mainly within the astrocytes [12]. In basal conditions, astrocytes release GDs producing a constant concertation of Guo in the extracellular milieu. Of note, the spontaneous release of GDs from the astrocytes is significantly higher than their adenine-based counterparts [16]. The transport of GDs (including Guo) into an extracellular compartment is mediated by equilibrative nucleoside transporters (ENT) [17]. Moreover, guanine nucleotides can undergo extracellular hydrolysis by membrane-bound ectonucleotidases, providing a secondary source of Guo in the extracellular milieu [18]. After the release into the extracellular compartment, Guo can be transformed into guanine (GUA) [19]. The phosphorolytic breakdown of guanosine to guanine is catalyzed by purine nucleoside phosphorylase (PNP), which is constitutively released by glial cells and neurons into the extracellular space [20,21]. Ultimately, guanine is deaminated by guanine deaminase forming xanthine [22,23].

### 2.2. Neurotrophic Effects of Guoanosine in CNS—Role in Neurogenesis, Neuritogenesis, and Cell Differentiation

GDs induce proliferative effects, emphasized by an increase in the number of neurons, and proliferation markers in both in vitro and in vivo studies [12,24,25,26,27]. These effects can be attributed to GD-induced soluble factor release (neuronal growth factor (NGF), transforming growth factor (TGF), fibroblast growth factor-2 (FGF-2), brain derived neutrophic factor (BDNF), and Guo-induced adenosine release [12,28,29,30]. In a study performed in vivo on a Parkinson’s disease model, Guo treatment increased progenitor/stem cell proliferation in the subventricular zone (SVZ) [24]. A subsequent study, performed on stem cells isolated from 1-day-old healthy rats, corroborated these observations, also reporting Guo-mediated neural stem cell (NSC) proliferation [28]. A more recent study presented that Guo is also able to induce the proliferation of NSCs sampled from adult mice [31].

MAPK and PI3K cascades are well studied molecular pathways involved mainly in neuronal proliferation, differentiation, and survival [32]. The protein kinase C (PKC) pathway takes part in synaptogenesis and, together with protein kinase A (PKA), regulates extracellular matrix (ECM) proteins [33,34,35]. A different signaling pathway, Ca2+/calmodulin-dependent protein kinase II (CaMKII), participates in Ca2+ signaling and mediates neuronal development and plasticity [36].

The molecular mechanism which would explain Guo-mediated trophic effects has not been fully unraveled. However, some studies indicate that the aforementioned molecular pathways are involved in Guo effect mediation [26]. Moreover, neuronal receptors, such as A2A, NMDA, and non-NMDA receptors, also contribute to the Guo-mediated proliferative and prosurvival effects observed in in vitro studies [25]. 

Guo stimulates also neuritogenesis—sprouting the neurites from the cell body forming connections between neurons. GTP and Guo coincubated with NGF were able to enhance the stimulatory action of NGF on neurite growth via distinct mechanisms [30,37]. More in-depth studies observed an increase of cAMP and activation of HO-1 and PRK1 in neurons in models of neuronal neurite arborization outgrowth [30,38,39]. HO-1 is an enzyme with antioxidant properties, whereas PRK1 is involved in actin cytoskeleton regulation and neuronal differentiation [40]. In SH-SY5Y neuroblastoma cells, a model of neuronal differentiation, Guo halted the cell cycle in neuroblastoma and promoted differentiation marker expression [41]. In line with this, a different study presented the Guo-elicited proliferation of NSC, followed by differentiation toward neurons [31]. 

GMP or Guo promotes the reorganization of astrocytic ECM proteins (laminin and fibronectin) in the neuron-astrocyte coculture model. The Guo/GMP-mediated ECM modulation is involved in the neuron-astrocyte interaction process and in trophic effect mediation. Furthermore, this study reported that Guo/GMP did not affect the process of neurogenesis. However, the number of neurons in cocultures increased, putatively due to increased neuronal viability or the neuritogenetic properties of laminin [26].

### 2.3. Guanine Derivatives and Neuroprotection

The first investigations into the role of GDs in neuropathologies found that the level of GDs is persistently elevated for 7 days after an ischemic injury [42]. Moreover, other studies showed that GDs are physiologically present in cerebrospinal fluid in nanomolar concentrations and rise by three- to fivefold within 30 min of hypoxic/hypoglycemic conditions [16,43]. Furthermore, it was demonstrated that under hypoxic conditions, the extracellular concentrations and activity of PNP decreased, which subsequently prolonged the presence of Guo in the extracellular compartment [21].

These discoveries prompted a new approach in establishing the role of GDs in CNS pathophysiology. It was suggested that GDs constitute an endogenous restorative system that activates after an injury, and its role is to prevent further damage and to re-establish tissue function [19]. Aside from the ischemic stroke, GDs also present a protective effect in in vivo models of other CNS disorders, such as epileptic seizures, Parkinson’s disease, Alzheimer’s disease, spinal cord injury, sepsis, gliomas, and hepatic encephalopathy [44,45,46,47,48,49,50,51,52,53]. It is important to highlight the fact that most of the neuroprotective effects observed in animal models were achieved using exogenously administered guanosine. Therefore, amongst other GDs, guanosine is the most promising potential therapeutic agent [45,46,47,48,49,50,51,52,54].

### 2.4. Guanosine-Specific Targets: Receptors and Binding Sites

It is still under debate if guanosine has its own specific receptor. Interestingly, as it was demonstrated in the animal models, guanosine probably has some kind of its own G-protein coupled receptor (GPCR). Guo binds strongly to this binding site, and naturally occurring purines (GDP, GMP, ATP, adenosine, xanthine, hypoxanthine, caffeine, theophylline) cannot displace guanosine from this location [55,56,57]. Moreover, it was demonstrated that this yet unknown binding site is different from well-characterized ARs [57,58]. Possible GPCR candidates include GPR174/LPS3 [59], which is highly homologous with P2Y10, and GPR23/LPA4, which shows high homology with human P2Y5 receptor [57,60]. Moreover, A_1_Rs and A_2a_Rs form receptor complexes with members of the P2Y receptor family. Additionally, P2Y5 receptors share high homology with GPR23. Thus, it is reasonable to hypothesize the occurrence of molecular interactions between GPR23 and A_1_R\A_2a_Rs [57,61]. Nevertheless, despite promising studies, the guanosine-specific receptor has not yet been fully characterized, and thus Guo remains an orphan neuromodulator.

Furthermore, Guo was identified as a weak A_1_ receptor (A_1_R) and A_2A_ receptor (A_2a_R) agonist [62,63,64]. It is still unknown to what extent these receptors are responsible for the neuroprotective effect of guanosine. Moreover, some studies show that the antioxidant effect is A_1_R dependent but A_2A_R independent [62,65,66,67]. This is contrary to the results of Dal-Cim et al., who indicate that some neuroprotective effects may be conducted via A_2A_R but not A_1_R [68]. Some studies suggest that it is the interplay between coexpressed receptors and the fine-tuning mechanism that are responsible for the neuroprotective effect [64,69].

Guo can target not only certain receptors but also potassium channels. Kir 4.1 is an inwardly rectifying K^+^ channel commonly found in glial cells in the CNS. This channel plays a role in the sustainment of extracellular K^+^ homeostasis, resting membrane potential, and regulation of glutamate uptake [70]. Chronic exposure to Guo in rat cortical astrocytes in vivo promoted the expression of Kir4.1. Of note, inhibition of the translational process prevented the Guo-induced upregulation of Kir 4.1, suggesting that the Kir 4.1 upregulation stimulated by Guo is achieved through de novo protein synthesis [71]. Moreover, Guo acts via large-conductance Ca^2+^-activated K^+^ (BK) channels [27,72]. In this case, guanosine binds to the alfa subunit of K^+^ channels and increases K^+^ conductance [73]. Moreover, Guo modulates NMDA receptors and probably glutamate transporters, including GLT-1. [74,75,76,77].

Overall, guanosine-mediated neuroprotective effects may be mediated by the interplay between known adenosine receptors and/or potential guanosine binding site as well as BK channels.

## 3. Key Pathophysiological Events of Ischemic Stroke and Targets for Guanosine

The main contributory factors involved in the pathophysiology of ischemic stroke are oxygen and glucose deprivation (OGD), reperfusion injury, glutamate excitotoxicity, and neuroinflammation. The brain is extremely vulnerable to ischemic damage, due to its high metabolic rate, limited energy storage capacity, and sole dependence on glucose as an energy substrate [78]. An area characterized by irreversible neuronal damage due to energy depletion is called an ischemic core. In the ischemic core, within minutes, cells undergo necrosis and excitotoxic cell death. Around the ischemic core is a functionally compromised but structurally intact tissue called the penumbra. In this area, cell death occurs at a slower rate, mostly through oxidative stress-mediated processes like apoptosis and inflammation [79]. As both of these mechanisms are triggered in a relatively orderly fashion, this opens up much more possibilities for therapeutic targeting compared to necrotic cells [80].

Reperfusion injury usually occurs in the course of poststroke thrombolytic therapy or thrombectomy, due to blood flow restoration in the previously occluded blood vessels. Counterintuitively, therapeutically achieved blood flow restoration brings detrimental consequences for the peri-infarct region as it contributes to a secondary burst of ROS generation [81].

Prolonged energy deficiency promotes a rise in glutamate (Glu) levels due to the increased release of Glu into the synaptic cleft and impaired Glu reuptake. Subsequently, excessive stimulation of NMDA receptors by glutamate results in Ca2+ and Na+ influx, which causes cell swelling and excitotoxic cell death [82]. The latter is a result of neuronal overstimulation and subsequent mitochondrial dysfunction, uncontrolled production of reactive oxygen species (ROS), and activation of proapoptotic pathways [83].

Shortly after ischemic stroke onset, the neuroinflammatory processes unfold [84]. The trigger for the acute phase of inflammation is damage-associated molecular patterns (DAMPs) which are released from dying and necrotic cells in the ischemic core. These molecules then activate local immune cells and perivascular endothelial cells by acting on Toll-like receptors and purinergic receptors [85], subsequently leading to inflammasome activation, which initiates a fully fledged inflammatory response [86]. The increased levels of chemokines cause chemotaxis of circulating leukocytes into the injury site. This process of infiltration is supported by activated microglia that upregulate adhesion molecules on cerebral vasculature [87,88,89]. Subsequently, the microglia (and other immune cells) produce metalloproteinases (MMPs) which increase BBB permeability, allowing other immune cells easier access to damaged brain areas [90]. Locally activated microglia and infiltrated macrophages can then perform proinflammatory or anti-inflammatory functions, depending on their molecular phenotypes [91].

On a molecular level, ischemic insult causes an upregulation of mitogen-activated protein kinases (MAPK) and an expression of the gene encoding nuclear factor kappa beta (NF-kb) protein complex [92]. NF-kb is a heteromeric transcription factor that most commonly is made up of p50 and p65 [93,94]. After translocation to the nucleus, NF-kb binds to specific sites of a DNA and induces transcription of proinflammatory cytokines and inducible NOS (iNOS), which is an enzyme responsible for increased ROS production and inflammation enhancement [95,96].

Most of the abovementioned aspects of neuroinflammation are detrimental to neuronal tissue and further deepen the ischemic injury. Thus, post stroke inflammation presents itself as a potential target of ischemic stroke treatment.

On a cellular level, the plethora of protective mechanisms of guanosine was studied in cortical slices or neural cells exposed to glucose-free medium and hypoxic atmosphere in a so-called oxygen-glucose deprivation (OGD) protocol [27,97]. OGD protocol is a well-established model of mimicking the most significant aspects of ischemic injury. Frequently, the OGD is followed by the reoxygenation period, which simulates the reperfusion stage of ischemic stroke [98].

### 3.1. PI3K, MEK, and PKC Are Involved in Guanosine-Mediated Neuroprotection

**Figure 1:**
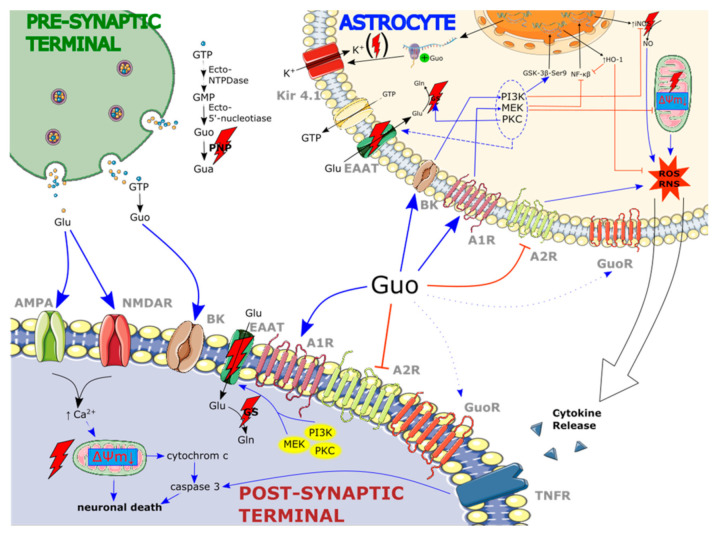
Overview of the most important mechanisms that contribute to guanosine-induced neuroprotection under ischemia: guanosine 5′-triphosphate (GTP) is released from the presynaptic terminal of neurons via synaptic vesicles but can also be transported directly from astrocytes into extracellular space. Extracellular guanosine nucleotides undergo hydrolysis by ectonucleotidases into guanosine 5′-monophosphate (GMP) and subsequently guanosine (Guo). The end product of guanosine nucleotide degradation is guanine. The cleavage of guanosine to guanine (Gua) is catalyzed by purine nucleoside phosphorylase (PNP), the concentration of which is decreased under hypoxic conditions (as marked by a red lightning bolt). Guanosine has a putative specific binding site (GuoR); however, its role has not been fully characterized (the putative Guo–GuoR interaction is marked as dotted arrows). Nevertheless, guanosine can act upon adenosine receptors (A_1_R and A_2A_R) and potassium big conductance channels (BK). Guanosine activates cellular molecular pathways, such as PI3K/Akt (PI3K), MEK/ERK (MEK), and protein kinase C (PKC). Activation of these molecular pathways leads to stimulation of amino acid transporters (EAATs) (depicted as a dashed arrow). Moreover, guanosine acting via the PKC pathway increases glutamine synthase (GS) activity which, combined with EAAT stimulation, protects against glutamate excitotoxicity. The physiological base activity of EAATs and GS is disturbed under ischemic conditions. Guanosine promotes protection against reactive oxygen species (ROS) and reactive nitrogen species (RNS) by activation of MEK and PI3K. These cellular pathways downregulate the expression of iNOS via NF-kb inhibition, upregulate expression of HO-1 via GSK-3β phosphorylation at Ser9 (GSK-3β-Ser9), and prevent loss of mitochondrial membrane potential (ΔΨm↓). Notice that some of these effects counteract the detrimental events that occur under ischemic conditions. These include mitochondrial membrane depolarization and iNOS upregulation. The consequence of increased ROS/RNS production is a release of proinflammatory cytokines (marked as an open arrow) some of which then act on tumor necrosis factor receptors (TNFR) promoting apoptosis in neurons. Chronic supplementation of guanosine prevents reduction of inwardly rectifying K^+^ channels (Kir 4.1) by promoting de novo Kir4. 1 synthesis. Importantly, expression of Kir 4.1 and/or Kir-mediated currents are reduced up to 14 days after an ischemic injury (marked as red lightning bolt put in braces). Figure designed using image template from Servier Medical Art https://smart.servier.com/image-set-download/, accessed on 12 March 2021.

Although the manner in which Guo exerts its effects is still not fully unraveled, a few signaling pathways have been discovered that play a role in Guo effects mediation. In 2008, Oleskovicz et al. demonstrated that Guo acts via the modulation of PKA, PKC, MAPK, and/or PI-3K pathways. These signaling pathways were blocked by specific inhibitors, resulting in the reduction of guanosine-induced neuroprotection [99]. Later studies further confirmed the involvement of these pathways in Guo effect mediation [27,68,100,101,102,103] (Figure 1).

Guo induces the phosphorylation of protein kinase B (PKB/Akt) via PI3K, which leads to the inactivation of GSK3β through phosphorylation at Ser9 [100]. In a study by Molz et al., the use of PI3K inhibitor (LY294002) prevented Guo-induced GSK3β phosphorylation. Moreover, incubating hippocampal slices with Guo presented a significant rise of GSK3β-Ser9 after 30 min of exposure [101]. In line with this study, blocking PI3K with a different inhibitor (wortmanin) also abolished Guo-induced PKB/Akt phosphorylation. Moreover, in the presence of a BK channel inhibitor (charybdotoxin), the phosphorylation of PKB/Akt promoted by Guo was also blocked [27]. These results indicate that the neuroprotection elicited by Guo involves BK channel activation, a subsequent PI3K-PKB/Akt pathway activation, and phosphorylation of GSK3β, which is a downstream effector of that pathway.

The MAPK/ERK pathway is another crucial pathway that, similar to PI3K, mediates a variety of Guo neuroprotective effects. Its contribution was presented in two studies conducted by Dal-Cim et al. on hippocampal slices and cortical astrocyte cultures. In the presence of MEK inhibitor (PD98059), Guo protection against OGD-induced damage was prevented. Moreover, a similar effect was achieved by blocking A1R. These results show that Guo exerts its neuroprotective effect via a mechanism that involves both the MAPK/ERK pathway and A_1_R activation [68,102]. In a different study, using a different MAPK inhibitor (SB203580) in glucose-deprived C6 astroglial cells, a similar effect was observed [103].

Several guanosine effects are also dependent on the PKC pathway. Blocking PKC with BIS II in C6 astroglial cells abolishes some of the neuroprotective effects of Guo [103]. Moreover, Guo coincubated with another PKC inhibitor (chelerythrine) lost the ability to prevent cell death in astrocytes from the murine cerebral cortex [102].

In summary, the most important molecular pathways that participate in Guo effect mediation are PI3K, MEK, and PKC. 

### 3.2. Guanosine-Mediated Neuroprotection Depends on BK Channels Activity

During an ischemic event, the expression of Kir 4.1 and/or Kir-mediated currents are reduced up to 14 days after an injury [104,105,106]. In a study from 2006 conducted by Befenati et al., chronic exposure to Guo in unexposed to OGD rat cortical astrocytes in vivo promoted the upregulation of Kir4.1 [71]. This study indicates that Guo could potentially counteract the effect of ischemic insult on K+ homeostasis. Nevertheless, further research must be conducted to support this thesis. 

In a study performed on hippocampal slices subjected to OGD conditions, the blocking of K+ channels with 4-aminopyridine (4-AP), a voltage-dependent K+ channel blocker, abolished Guo-induced neuroprotection [99]. Moreover, further research showed that Guo effects are mediated by BK channels (BK) [27,72]. BK channels are determined to facilitate membrane potential and activate the PKB/Akt pathway which acts as a cellular defense against oxidative damage [72,107,108,109]. Charybdotoxin, a BK selective blocker, was able to impede Guo-induced neuroprotection during the reoxygenation period [27]. In line with this study, charybdotoxin also blocked the protective effects of Guo on SH-SY5Y cells against mitochondrial oxidative stress [72]. In the light of the reported evidence, it is conceivable that BK channels are an essential part of the neuroprotective molecular cascade initiated by Guo.

### 3.3. Guanosine Acts Against Glutamate Excitotoxicity

One of the major mechanisms implicated in guanosine neuroprotection is the stimulation of the glutamate uptake, thus counteracting glutamate excitotoxicity [13,75,110,111]. Primarily, Guo protection against glutamate toxicity was studied based on in vitro glutamate excitotoxicity and seizures models [112,113,114]. During hypoxia/ischemia, a rapid increase of glutamate occurs mainly due to impairment of the glutamate uptake system, the release of excessive glutamate, the reversal of glutamate transporters activity (reverse uptake), and decreased activity of glutamine synthetase (GS) [115,116,117]. Using the model of hippocampal slices subjected to OGD with subsequent reoxygenation, it was demonstrated that guanosine administration promoted glutamate uptake by increasing the uptake Vmax and prevented the reversal of uptake induced by excessive glutamate [27,99,111,118]. Notably, the stimulatory effect of guanosine on glutamate uptake was predominantly observed in the reoxygenation but not in the hypoxic period, even when administered up to 3 h in the reoxygenation period [27,97]. This particular effect was caused by ATP level depletion during the ischemic period of OGD, which caused the blockade of ATP-dependent glutamate uptake (relying on Na–K–ATPase activity) [119,120]. The mechanism underlying the ability of guanosine to promote glutamate uptake relies mostly on modulating the glutamate transporter 1 (GLT-1) activity and restoration of GLT-1 expression to basal levels under OGD but also indirectly on the restoration of GS activity to physiological levels, most likely through the activation of the PKC pathway [68,75,102,103,121].

Regarding the intracellular signaling pathways involved in guanosine-mediated glutaminergic modulation, it was demonstrated that guanosine acting through the activation of A1R and the blockade of A2AR receptors or the putative Gi protein-coupled signaling site recruited PI3K/protein kinase B(Akt) and, predominantly, the MEK/ERK and PKC pathways [68,102]. MEK/ERK signaling cascade involvement in astrocytes is particularly important since the activation of ERK1/2 cascade protein is directly related to the activity of glutamate receptors [68,122]. The activation of PI3K/Akt by the Guo signaling cascade was observed only in hippocampal slices subjected to OGD but not in cortical astrocyte cultures, suggesting that the activation of this antiapoptotic, glutamate modulatory pathway by Guo is cell-type specific and may promote PI3K-dependent glutamate uptake in neurons through excitatory amino acid carrier 1 (EAAC1) [102,123].

### 3.4. Guanosine Prevents Mitochondrial Dysfunction

On the molecular level, the impairment of mitochondria results in increased reactive oxygen species (ROS) production, redox homeostasis disruption, and activation of apoptotic cell death [124,125,126]. The main events that contribute to mitochondrial dysfunction are excess Ca2+, mitochondrial swelling, loss of mitochondrial membrane potential, impaired oxidative phosphorylation, and accumulation of ROS [127]. Mitochondrial membrane depolarization is a well-known marker of mitochondrial dysfunction [126]. The loss of mitochondrial membrane potential is followed by an increase in ROS production with subsequent oxidative damage, reduced ATP production, and redox homeostasis disruption [128].

Several studies have postulated that Guo can prevent mitochondrial damage by direct ROS scavenging and/or via activation of molecular pathways that induce antioxidant effects [103,107]. More recent studies vastly diminished the role of Guo as a direct antioxidant, showing no or little involvement in nitric oxide (NO) scavenging activity [127,129]. Therefore, the mechanism of action which is responsible for the Guo effect on mitochondria is probably related to its ability to activate molecular pathways which then elicit antioxidant effects. In a study conducted by Dal-Cim et al., performed using hippocampal slices, blocking the MAPK/ERK pathway abolished Guo-induced protection against mitochondrial membrane depolarization. Moreover, blocking A_1_R also removed the Guo effect on membrane potential. These results suggest a significant role of A1R and MEK in the mediation of Guo-induced protection against mitochondrial dysfunction during OGD [68].

In the most recent study conducted by Courtes et al. using liver mitochondria, Guo demonstrated a protective effect against Ca2+-induced mitochondrial dysfunction [127]. Interestingly, Guo improved mitochondrial function without any link to the stabilization of mitochondrial membrane potential or direct ROS scavenging. In this study, Guo prevented in vitro Ca2+-induced mitochondrial impairment by reduction of mitochondrial swelling and ROS levels. Moreover, Guo boosted mitochondrial metabolism and helped to establish energy homeostasis [127].

It is important to highlight that the potential differences between liver mitochondria and mitochondria found in CNS could undermine the significance of these data in terms of stroke. Moreover, the heterogeneity of mitochondria within CNS itself also implies caution with the interpretation of data obtained from different brain regions [130,131,132].

### 3.5. Guanosine Mediates the Decrease in NO Overproduction

NO is an important CNS messenger and neurotransmitter that actively participates in many pathological processes that occur during an ischemic stroke [133,134]. In an early response to ischemia, NO released from endothelium exerts protective effects by promoting, e.g., vasodilation. However, soon after, NO is massively overproduced by neurons and glia, and its effect becomes deleterious to the surrounding tissues [135]. In neurons, NO is produced mainly by constitutively expressed neuronal nitric oxide synthase (nNOS). Contrarily, in glial cells, the dominant isoform of NOS is iNOS. In contrast to nNOS, iNOS is upregulated in response to OGD, excessive ROS production, and glutamate excitotoxicity [101,136,137,138].

In line with several papers, Guo exerts its antioxidant effects by inhibition of NOS, downregulating the production of NO and consequently lowering levels of reactive nitrogen species (RNS) and ROS, which in turn ameliorates cell viability [38,68,129].

Studies performed on hippocampal slices and C6 astroglial cells reveal that Guo hinders the expression of iNOS by inhibition of NF-kb, more precisely by preventing it from binding to the promoter sequence of iNOS. Interestingly, the aforementioned Guo effect can be diminished by blocking either A_1_R or signaling pathways like MEK or PI3K [68,107]. Moreover, the Guo downregulating effect on NF-kb activation may be mediated by heme oxygenase 1 (HO-1), since blocking this enzyme does not prevent NF-kb from raising in the presence of Guo in OGD conditions [107]. Interestingly, although Guo can elicit iNOS suppression at a concentration of 100 µM, it does not present this ability at concentrations of 30 or 300 µM [101].

It has now been hypothesized that Guo’s effects on NO production may be mediated not only by iNOS but also by nNOS. In hippocampal slices subjected to OGD, Guo can in fact decrease iNOS induced by OGD. However, selective blocking of iNOS did not elicit a decline in RNS levels. Contrarily, in the presence of nNOS inhibitors, NO and ONOO- levels significantly decreased [129]. Thus, the modulation of nNOS rather than iNOS activity by Guo can bring antioxidative aid to damage induced by RNS.

More recently, a study on hippocampal slices subjected to OGD followed by reoxygenation presented a modulatory effect of Guo on NO levels. Guo coincubated with the nonselective NOS inhibitor (L-NAME) prevented a decline in ATP production, lactate release, and glutamate uptake in murine brain slices. However, in the presence of NO donors (DETA-NO or SNP), the protective effect of L-NAME or guanosine on bioenergetics and glutamate clearance was abolished [139].

Maintaining sustainable ATP concentration levels within the cell is an important task that can vastly increase the chance of survival in the OGD environment [140]. In CNS, several studies have proven the importance of astrocytes in the maintenance of neuronal energetic equilibrium. In astrocytes subjected to oxygen scarcity, oxygen-independent metabolic pathways such as glycolysis are activated. The end product of glycolysis lactate is then consumed by neurons providing them with energy [141,142]. During the OGD period, extracellular lactate levels decrease due to a putative increase in lactate consumption by neurons enduring hypoxia [143,144]. Guo was able to increase lactate availability and ATP levels in ischemic hippocampal slices. Interestingly, a similar effect was achieved using L-NAME, showing that Guo can alter cellular bioenergetic metabolism putatively via a mechanism involving NO level modulation [139].

In summary, Guo acts through cellular pathways and receptors that modulate NOS enzymes expression and subsequently NO levels. This in turn provides a decrease in ROS and RNS production and prevents the disruption in cell bioenergetics promoted by experimental ischemic stroke models.

### 3.6. Guanosine Exerts Antioxidative Effects through the Activation of Heme Oxygenase-1

HO-1 is an enzyme involved in the breakdown of pro-oxidant heme into antioxidative bilirubin and biliverdin, consequently shifting the balance in favor of antioxidants [145]. Moreover, HO-1 is also involved in the mediation of anti-inflammatory and antiapoptotic effects [47,146]. Mounting evidence suggests that the activation of HO-1 is one of the cell’s rudimentary antioxidant defense [38,147]. Of note, in astrocytes subjected to oxidative or/and inflammatory injury, HO-1 is upregulated, counteracting the insult [107,148]. Considering the fact that an inseparable component of ischemia is an increase in ROS production and neuroinflammation, HO-1 has the potential to be targeted by future neuroprotective drugs [47,79,83,84,107].

The most notable function of HO-1 is intracellular redox environment maintenance, achieved by blocking the activity of iNOS. Moreover, HO-1 can inhibit NF-kB translocation from the cytoplasm to the nucleus, preventing it from inducing the production of inflammation mediators such as IL-1, TNF-alpha, iNOS, and cyclooxygenase-2 (COX2) [107,149]. Azide is a well-known respiratory chain inhibitor and is commonly used to evoke oxidative and nitrosative stress in experimental models [150]. In a study conducted by Quincozes-Santos et al. performed on C6 astroglial cells, Guo presented antioxidative properties by counteracting the detrimental effects of azide-induced ROS production [107]. In line with this, a different study showed that the inhibition of HO-1 by Sn(IV) protoporphyrin-IX dichloride (SnPP) abolishes the protective effects of Guo against mitochondrial stress. Additionally, this study also revealed that HO-1 activation induced by Guo may be preceded by PI3K/Akt/GSK-3beta pathway activation [72].

In summary, guanosine acting via HO-1 modulates the activity of many proinflammatory and pro-oxidant mediators. These include NO, iNOS, TNF-alpha, IL-1, COX2, and NF-kB.

### 3.7. Guanosine and Post-translational Processes in Ischemia—The Potential Role of SUMOylation

Most recently, guanosine was implicated in modulating the SUMOylation by interacting with A1 and A2A receptors [151]. SUMOylation is defined as a type of post-translational modification mediated by a small ubiquitin-like modifier peptide, which covalently binds to lysine residues of specific proteins, analogously to the ubiquitination process [152,153]. SUMOylation plays important physiological roles including synaptic maturation, regulation, and plasticity [154]. Under hypoxic/ischemic conditions, increased protein SUMOylation takes part in the endogenous neuroprotective system [155,156,157]. It was clearly demonstrated that extracellular, exogenous guanosine increases global protein SUMOylation in cortical astrocytes and cortical neurons. However, the effect was observed only up to 1 h after guanosine stimulation, thus suggesting that at longer time points, the effect of guanosine is eventually counteracted by deSUMOylating enzymes [151].

Together, this evidence suggests that guanosine is a SUMOylation enhancer, which may partially account for its neuroprotective effects under ischemia.

## 4. Protective Effects of Guanosine against Ischemic Stroke: Evidence from In Vivo Studies

In recent years, a number of in vivo studies confirmed the neuroprotective effects of guanosine after administration in the acute and chronic phases of ischemic stroke. The authors of these studies undertook different approaches to guanosine administration and studied diversified pathophysiological aspects of the ischemic stroke mechanism. Therefore, it is worth consolidating the existing knowledge to better depict the full spectrum of the neuroprotective effects of guanosine (Table 1).

### 4.1. Safety and Pharmacokinetics of Exogenous Guanosine in Rodent Models—Implications in Ischemic Stroke

Future neuroprotectants used in stroke treatment should have a wide therapeutic window, be rapidly distributed into the CNS, and target all of the brain components, including neurons, glia, and the BBB. Moreover, any drug’s efficacy should be unaffected by sex-specific differences and bring a low risk of side effects and interactions with other drugs (drugs used in the stroke treatment or patients’ daily medications). 

The drug’s bioavailability and the time in which the neuroprotectant reaches the penumbra are largely dependent on the administration route. After intraperitoneal (i.p.) administration, Guo crosses the BBB via nucleoside-specific transporters located at the endothelium of brain blood vessels, enabling Guo to reach the brain via systemic circulation [158]. Intraperitoneal (i.p) administration of exogenous Guo and GMP elicits a threefold increase of Guo in CSF after 30 min. Additionally, after 5′-nucleosidase inhibitor (AOPCP) administration, the GMP/Guo relative proportion altered in favor of GMP [159]. In line with this, i.p administration of Guo caused an increase in guanine, a direct Guo metabolite, after 30 min in a spinal cord sample [51]. Moreover, chronic administration of guanosine for 6 weeks resulted in an elevation of Guo metabolite levels in the CSF and plasma. Interestingly, due to Guo-induced adenosine release, the plasma adenosine level was also increased [160]. The results of the aforementioned studies confirm the metabolism of Guo in the central nervous system (CNS) and systemic circulation. Thus, the half-life of Guo is relatively low. An interesting approach to exogenous Guo administration was presented by Ramos et al. in 2016. In this study, Guo was administered intranasally (IN), therefore bypassing systemic circulation and going directly into the brain via olfactory and trigeminal nerves. By partially omitting systemic circulation, a smaller amount of Guo undergoes systemic metabolism. This, in turn, can raise the amount of Guo reaching the brain, consequently increasing the effectiveness of the administered dose. Moreover, when using the IN administration route, beneficial effects, such as the prevention of behavioral impairment and decrease of brain infarct volume, can be achieved with a dose seven times lower compared with using the i.p. administration route [161]. Furthermore, with this route of administration, exogenous Guo can reach the brain within 5 min compared to 15–30 min when administered i.p. [161,162]. A more recent study corroborated these results by outlining the increased time window in IN compared to the systemic route of administration. In the rat ischemia model (thermocoagulation of pial vessels), IN administration of Guo was able to attenuate neurological deficits when administered as late as 3 h after ischemia onset. Contrarily, using the systemic route, Guo-elicited neuroprotection could only be observed when administered immediately after stroke induction [163].

Guo-mediated neuroprotection comprises neurons, glia, and, as recently discovered, the BBB [63,121,160]. However, the main targets of Guo are the astrocytes [102,164]. Moreover, Guo modulates processes, including neuroinflammation and microglia activation, which can be detrimental to virtually all cells found in the brain [84,87,90]. Thus, together with evidence of Guo affecting remote areas from the ischemic lesion, it can be theorized that Guo-mediated neuroprotective effects are expressed globally throughout the CNS [163].

To our knowledge, no studies have reported any major side effects of exogenously administered Guo in in vivo models [74,163,165]. Guo administered i.p did not impair renal function. Indeed, there is evidence of Guo-mediated renoprotection [166]. However, doses higher than 240 mg kg^−1^ caused an elevation in liver function biomarkers. Moreover, Guo significantly decreased barbiturate-induced sleeping time in rodents [74,167]. Interestingly, as opposed to classic NMDAR antagonists (MK-801, ketamine), Guo most probably does not induce psychomimetic effects [54]. Nevertheless, Guo is well known for inducing amnestic effects in rodents [168,169]. This effect is probably the result of the inhibitory action of Guo on the glutaminergic system in the brain limbic structures [170]. Guo presents strong advantages over adenosine, a nucleoside with similar neuroprotective properties, in regard to adverse effects. Compared to adenosine, Guo has much less impact on basal arterial blood pressure [171]. Nevertheless, in the situation where Guo pretreatment is combined with adenosine infusion, the enhancement of the Guo-mediated adenosine’s effects can provoke cardiovascular shock [171]. 

Only one work to date studied the differences in Guo treatment effects between male and female in vivo models, presenting better sensorimotor long-term recovery in female rats [172]. Nevertheless, further research has to be conducted to distinguish the sex-specific differences in response to Guo from neuroprotective effects induced by estrogens [173].

### 4.2. Neuroprotective and Neurorestorative Effects of Guanosine in Rodent Stroke Models

The neuroprotective effect of guanosine is time and dose dependent. The guanosine administration protocols were evaluated for the first time in models of neonatal hypoxic-ischemic (HI) injury and chronic cerebral hypoperfusion [160,174]. Guanosine administration up to 6 h following HI injury in three consecutive doses was found to modulate the glutamate uptake, thus preventing glutamate excitotoxicity [97,174]. Importantly, only the three-dose administration protocol was sufficient to achieve the protective effects of guanosine, regardless of whether the first dose was given immediately, 3 h, or 6 h after HI, indicating the dose-dependent effect of guanosine [174].

The described protocol was later adopted in ischemic stroke rodent models. The systemic administration of guanosine directly (t = 0 min) or within the first 6 h after permanent focal ischemia or middle cerebral artery occlusion (MCAo) with consecutive doses administered hours to days postischemia was found to increase the postischemic survival of rats and significantly decrease the infarct volume up to 40% [175,176,177,178]. The Guo-treated animals achieved progressive improvement in sensorimotor performance and partial restoration of motor dysfunctions assessed by the neurological deficit scale (NGS), indicating both the neuroprotective and neurorestorative properties of guanosine. Interestingly, the Guo-mediated neuroprotective effects differed between sexes. It was demonstrated that the female rats were more sensitive to Guo administration and reached significantly better long-term improvement in terms of sensorimotor deficits, independently of the estrous-cycle phase [172]. A subsequent set of studies demonstrated that the systemic administration of Guo 30 min before MCAo combined with postischemia Guo administration resulted in the most considerable decrease in infarct volume and restoration of neurological function compared to the administration at t = 0 min [176].

The Guo-mediated mechanism of neuroprotection observed in rodent models of the acute phase of ischemic stroke stands in agreement with in vitro studies [60,99,179]. Ex vivo studies performed 24 h after permanent focal ischemia in Guo-treated animals demonstrated the Guo-mediated restoration of decreased GLT-1 expression and increase in glutamine synthetase (GS) activity, thus increasing intracellular glutamate uptake [121]. Guo undeniably protects against glutamate excitotoxicity, but its protective effects observed in vivo also depend on antioxidative and anti-inflammatory properties [47,121,177]. Ischemic stroke triggers a massive production of reactive ROS and RNS, which causes oxidative stress response and an increase in expression of antioxidant enzymes (SOD, CAT) [96,180,181]. However, the activity of antioxidant enzymes drastically decreases, emphasizing the inefficiency of the physiological redox homeostasis system under severe oxidative stress [180,182]. Guo treatment was found to decrease lipid peroxidation and prevent the increase of NO and ROS levels. Moreover, Guo administration fully restored the decreased activity of SOD and CAT, increased the expression of SOD, and partially restored vitamin C levels [177]. A later also study revealed the strong anti-inflammatory properties of Guo. Guo administered in the acute phase of ischemic stroke suppressed the activation and infiltration of microglia to the periphery of the ischemic core. These results were supplemented by a Guo-mediated decrease in proinflammatory cytokines (IL-1, IL-6, TNF-α, IFN-γ) and prevention of a decrease of anti-inflammatory IL-10 cytokine in the CSF and ischemic lesion periphery, consequently restoring the pro-/anti-inflammatory balance [121]. 

The intranasal administration of Guo was studied in models of permanent focal cerebral ischemia [161,163]. One of the major advantages of IN over the systemic administration route was the rapid penetration to the CNS (5 min post-ischemia), wide distribution in the CSF, decreased influence of systemic metabolism, and lower effective dose. [161]. However, IN Guo administered within 1–3 h postischemia was ineffective in infarct volume reduction. Remarkably IN Guo reduced mitochondrial dysfunction in the penumbra, which positively correlated with neurological outcome [161]. This observation is in line with the correlation of a decrease in oxidative stress markers and sensorimotor recovery observed after systemic administration of Guo [121,178]. Recently, a study by Müller et al. reinforced the safety and long-lasting neuroprotective effects of intranasal Guo. IN Guo administration up to 3 h after ischemic insult improved short-term, poststroke motor deficits and promoted long-term recovery. Using quantitative EEG (qEEG) it was demonstrated that Guo caused a decrease in the global state of synchrony (hyperexcitability) in both hemispheres, thus improving the functional state of the brain affected directly by ischemic insult and its more distant parts. Notably, the authors have shown for the first time that Guo partially prevents the disruption in BBB integrity induced by stroke. The exact nature of the interaction between Guo and BBB is not fully understood but may include the modulation of the survival/apoptotic PI3k/Caspase 3 pathway [163].

Additionally, the effects of Guo were also studied in the rodent model of MCAo followed by 5.5 h reperfusion. Guo administration 5 min before reperfusion or up to 30 min postreperfusion resulted in a dose-dependent decrease in infarct volume and neurological improvement [178]. The 16 mg/kg dose of Guo decreased the infarct volume by approximately 85%, whereas a concentration of 8 mg/kg resulted in a decrease of nearly 60%. Therefore, the dose dependence of Guo in a model of reperfusion is more expressed compared to permanent MCAo models of stroke, where there was no statistical difference between doses of 8 mg/kg and 16mg/kg [176], thus implying the differences in the neuroprotective mechanisms of Guo in permanent ischemia and reperfusion injury. In contrast to the ischemic period, the in vivo neuroprotective effect of Guo in reperfusion injury does not depend on the activation of ER stress pathways, prosurvival modification of calcium homeostasis, or modulation of glutamate uptake [178,183]. Interestingly, there were no observed sustained increases in prosurvival cysteine protease m-calpain levels compared to previous models of permanent MCAo, in which a Guo-mediated increase in m-calpain was implied in protection against necrotic/apoptotic cell death [176,184].

The administration of Guo in a narrow time window directly after or shortly before ischemic insult could be extremely challenging in the clinical environment. Therefore, delayed administration of guanosine after ischemia was studied in a rodent model of photothrombotic stroke (PT) [185]. Administration of guanosine 24 h after ischemic insult induced by PT resulted in remarkable improvement in neurological outcome, starting from 14 days poststroke. However, Guo’s treatment failed to reduce the infarct volume measured on day 7. Additionally, no significant neurological improvement in the acute phase was observed. Delayed Guo administration enhanced the endogenous neural progenitor cell proliferation in the peri-infarct region. This observation was also supported by the Guo-mediated major increase in BDNF and VEGF in the ipsilateral hemisphere after PT [185]. BDNF—a major traditional neurotrophic agent—and VEGF—a crucial endothelial growth factor—both play a pivotal role in the poststroke interplay between angiogenesis and neurogenesis by stimulating neuronal plasticity and enhancing neural stem cell (NSCs) migration [130,186,187]. Notably, the events of poststroke neurogenesis and angiogenesis are tightly linked and mutually regulated. In other words, the process of post-stroke vessel formation in the peri-infarct region enhances neuroblast adhesion and migration, while the secretion of VEGF by activated endothelial cells and NSCs acts both as an angiogenesis-stimulating factor and a strong neurotrophin [186]. Together, this evidence suggests that Guo promotes poststroke neurogenesis and angiogenesis, and its effect is neurorestorative rather than neuroprotective when administered in a delayed time interval. This observation also reinforces the concept of the neuroregenerative and neuritogenic effects of extracellular Guo observed in vivo and in neural cell lines [19,31,66,188].

## 5. Current Challenges and Limitations of Guanosine Application in Ischemic Stroke

The pathophysiological processes underlying stroke are driven by the complex interactions between neurons, glial cells, vasculature, immune cells, leukocytes, and matrix components, all participating in the processes of brain injury and neuroregeneration. Currently, the neuroprotective mechanisms of guanosine have been studied mainly concerning pathophysiological processes occurring in neurons and glial cells: for instance, glutamate excitotoxicity or neuroinflammatory response. However, the interactions between guanosine and other components of ischemic stroke are poorly understood. Therefore, we propose to investigate: (i) the effect of Guo on ischemia-induced endothelial activation; (ii) the relationship between Guo and BBB permeability and integrity; (iii) the properties of Guo in the context of peripheral immune cell activation and infiltration to the ischemic area; and (iv) the interactions between guanosine and extracellular purine nucleoside phosphorylase (PNP).

Based on the most recent evidence, the Guo mechanism of action is tightly connected to interactions with cell-surface adenosine receptors. Guo effects are mediated by A1 activation and negative modulation of the A2AR receptor. Moreover, Guo requires both A1R and A2AR coexpression in the form of the A1R-A2AR heteromer [189]. Adenosine receptors are broadly distributed in brain vessels, platelets, and neutrophilic granulocytes and regulate every step of endothelial-related inflammatory processes and vasodilatation [190,191]. Therefore, Guo, through interactions with AR and the proposed guanosine–adenosine interactions, may mitigate the endothelial activation and improve the cerebral microcirculation and thus the oxygen and substrate supply to the ischemic tissue after recanalization. However, this concept requires further evaluation, for instance, in models of in vitro endothelial cell cultures subjected to OGD.

Furthermore, the interaction between Guo and the BBB requires further evaluation. It was demonstrated that Guo can penetrate the BBB after systemic or intranasal administration most probably through equilibrative nucleoside transporters (ENT) [121,163]. Most recently Muller et al. introduced the concept of the in vivo modulation of BBB integrity by Guo [162]. This observation is especially important because ischemic stroke augments the BBB permeability and promotes the entry of immune cells and soluble inflammatory macromolecules into the CNS, thus aggravating the cellular response initiated by ischemia [192]. Moreover, the observed effect is reciprocal to adenosine interactions with BBB, which promote BBB permeability by signaling through A_1_ and A_2_ ARs [190,191]. Future studies may evaluate in detail the interactions between Guo and BBB and their dependency on Guo-mediated A_1_ activation and negative modulation of the A_2_AR receptor.

After an acute stroke, multiple immune cells systematically enter the brain parenchyma. Shortly after an ischemic insult, there is a rapid increase in microglia and peripheral immune cells (including dendritic cells, monocytes/macrophages, and neutrophils) that infiltrate within 1–7 days poststroke, resulting in further neuronal damage [193]. Concerning Guo, it was able to suppress the activation of microglia and the infiltration of polymorphonuclear granulocytes and monocytes/macrophages into the ischemic brain region, induced by the breakdown of the BBB [121]. However, the exact nature of this interaction remains elusive, and it is unclear whether it depends on the direct interaction between Guo and the BBB or a receptor-mediated interaction with peripheral immune cells, regarding the fact that neutrophils, macrophages/monocytes, and lymphocytes express a full spectrum of AR, presenting an accessible target for modulation by Guo [190,194]. On the contrary, Guo also activates specific subtypes of Toll-like receptors, TLR 2 and TLR 4, which take part in the activation of the immune system [195,196]. These possibly conflicting mechanisms should be evaluated in the context of interactions between Guo and cellular components of the immune system in ischemic stroke. 

Another limitation is that the current understanding of interactions between extracellular PNP and guanosine is incomplete. Astrocytes, microglia, and cerebellar granule neurons constitutively release the intracellular PNP to the extracellular compartment, which rapidly depletes the extracellular pool of guanosine [20]. Apart from this, there is a high activity of PNP in blood plasma [22]. PNP may be also released from lysed erythrocytes, for instance, during clot lysis in the cerebral artery. If so, the activity of extracellular PNP may reduce the pool of bioavailable exogenously administered Guo, thus hampering Guo from entering the penumbra in the CNS. This issue should be addressed in the future. One of the possible solutions to this issue may include the administration of PNP inhibitors together with Guo. 

## 6. Clinical Perspective

Thrombectomy and thrombolysis are the treatments of choice for ischemic stroke. Both are focused on the elimination of the direct cause of ischemic damage, i.e., the blood clot occluding a specific blood vessel. The most recent advances in this category are extended thrombolysis and thrombectomy protocols, which enable patients to benefit from these treatment options far beyond the standard time window. However, clinical observations show a vast diversity of outcomes found in patients with the same vessel occlusion and treated within a similar time window. These observations indicate that the current treatment methods are often insufficient and that many pathological processes that drive postischemic deterioration are beyond the scope of contemporary stroke patients’ care. We propose the concept of Guo-based therapy as a pharmacologically achieved neuroprotection, which would supplement recanalization-oriented treatment. The effects of Guo in such treatment regimens can be bidirectional. First of all, Guo administered in a short-time interval can directly rescue the tissue at risk in the penumbra from the effects of inflammation, oxidative stress, and glutamate toxicity and notably ameliorate the reperfusion injury caused by recanalization. Secondly, Guo can amplify and augment endogenous processes of neuroplasticity and neuroregeneration to support recovery and reduce the rate of poststroke complications.

## 7. Conclusions

In the present article, we comprehensively summarized the recent advances in the neuroprotective effects of Guo in ischemic stroke. Guo is a potent physiological neuromodulator, which takes part in a “backup” endogenous, restorative system, which protects neural cells from consequences of ischemia/hypoxia. Guanosine is a pleiotropic neuroprotectant in ischemic stroke: in the acute phase of ischemic stroke, Guo-induced neuroprotection is facilitated via antioxidative and anti-inflammatory actions putatively targeted against the oxidative stress and neuroinflammation elicited primarily by hypoxia and secondarily by reperfusion injury. In the chronic phase of ischemic stroke, Guo promotes poststroke neurogenesis and angiogenesis, thus stimulating neuronal plasticity and restoring neuronal function. Guo remains an orphan modulator; thus, its neuroprotective effects are mediated mainly by interactions with AR, glutaminergic receptors/transporters, and ionic channels involved in the process of anoxic depolarization. Furthermore, Guo activates the prosurvival pathways, most notably the MAPK signaling module, which takes part in the regulation of multiple modalities involved in oxygen sensing. Moreover, data collected through studies exploring guanosine’s pharmacokinetic properties also look promising. Guo can reach the CNS via systemic, oral, and intranasal routes, causing little to no side effects. This is the major advantage of Guo over adenine-based purines, specifically adenosine, which causes decreased heart rate, blood pressure, and sedation after systemic administration. Nevertheless, due to the short half-life of Guo, the perfect method of administration is still under debate. Overall, based on current evidence, future studies should unravel the precise mechanisms related to the neuroprotective effects of Guo concerning the neurovascular unit and the long-term effect of Guo administration. To address the complex machinery involved in the promotion of ischemic damage in the penumbra, a pleiotropic agent acting on many different pathophysiological processes is greatly desirable. We propose further evaluating the therapeutic potential of Guo in ischemic stroke supportive treatment in human participants.

## Figures and Tables

**Table 1:** The comparison of in vivo studies evaluating the neuroprotective effects of guanosine in ischemic stroke models focused on proposed molecular mechanisms and final outcomes.

Experimental Animal	Experimental Model	Route of Administration	Proposed Mechanism/s of Neuroprotection	Outcome/Guanosine Mediated Effects	Reference
Adult male Wistar Rat	MCAo	I.p.		Significantly smaller infarct volume in Guo group compared to control. Major improvement in gait and spontaneous activity in Guo group. No difference in number of cells undergoing apoptosis in penumbra region compared to control.	Chang et al. (2008) [175]
Adult male Sprague Dawley rats	MCAo	I.p.	Guo-induced increase in m-calpain level, preventing the necrotic cell death in ischemic area.	Significant decrease in infarct volume after 3 days. Significant decrease in infarct volume 6 h after preconditioning with 4mg/kg of Guo. Improvement in motor deficits on day one, two and three after Guo treatment. Increase in m-calpain level in ischemic area.	Rathbone et al. (2011) [176]
		Intracortical Injection		Significant reduction of infarct volume in Guo group.	
Adult male Sprague Dawley rats	MCAo with reperfusion	I.p.	Guo-induced inhibition of proinflammatory events induced by reperfusion. Inhibition of IL-8 release.	Time and dose-dependent significant reduction of infarct volume after reperfusion period. Decrease in infarct volume after 24 h following preconditioning 5 min prior to reperfusion. Narrow therapeutic window of Guo administration between 0 and 30 min after reperfusion.	Conell et al. (2013) [178]
Adult male Wistar Rat	Focal thermocoagulation in motor and sensorimotor cortices	I.p.	Guo-induced modulation of oxidative stress response system. Guo-induced glutamate uptake and intracellular conversion to glutamine.	Partial recovery of impaired limb function in cylinder test after 24 h, maintained for 15 days. Significant decrease in infarct volume. Prevention of ROS and NO levels increase in ischemic area. Increase in SOD and expression and activity. Increased CAT activity. Restoration of GLT -1 expression. Increased GS activity in ischemic region. Partial reversal of decreased vitamin C level.	Hansel et al. (2014) [177]
Adult male Wistar Rat	Focal thermocoagulation in motor and sensorimotor cortices	I.p.	Guo-mediated restoration of anti-/proinflammatory balance, prevention of inflammatory cell infiltration.	Significant improvement in motor performance in cylinder test, maintained for 15 days. Significant decrease in infarct volume and decrease in number of degenerated cells in penumbra after 24 h. Reduced infiltration of microglia and peripheral immune cells in the periphery of ischemic lesion. Decrease of IL-1, IL-6, TNF-α, and IFN-γ levels. Increase of IL-10 levels.	Hansel et al. (2015) [121]
Adult male Wistar Rat	Focal thermocoagulation in motor and sensorimotor cortices	I.n.	Guo-mediated improvement of mitochondrial status in penumbra.	Intranasal Guo administration providing almost immediate (5 min) delivery to the CNS, presenting higher CSF Guo concentrations compared to systemic administration. Significant improvement in symmetry rate in cylinder test. Decrease in infarct volume after 48 h only, when Guo administered immediately after ischemia. Significantly reduced mitochondrial dysfunction in penumbral region after Guo treatment 3 h postischemia. Correlation between mitochondrial status and motor performance of rats.	Ramos et al. (2016) [161]
Adult male C57BL/6J wild-type mice	PT	I.p.	Guo-induced increase in VEGF and BDNF enhancing poststroke angiogenesis and neurogenesis.	No decrease in infarct volume after delayed (24 h) administration of Guo. Significant improvement in forelimb function 14 and 28 days postischemia. Proliferation of neural progenitor cells and enhanced differentiation into mature neural cells at all poststroke time intervals. Increased angiogenesis in peri-infarct area. Increased expression of VEGF and BDNF in ischemic brain at 14 days postischemia.	Deng et al. (2017) [185]
Adult female and male Wistar Rat	Focal thermocoagulation in motor and sensorimotor cortices	I.p.		Full improvement in forelimb function of female rats in cylinder task, after Guo administration observed earlier in estrogenous group in comparison to only partial (60%) recovery in male subgroup.	Teixeira et al. (2018) [172]
Adult male Wistar Rat	Focal thermocoagulation in motor and sensorimotor cortices	I.n.	Guo-mediated prevention of disruption in BBB integrity.	Partial recovery of impaired limb function in cylinder test directly after ischemia in Guo treated group. Long term improvement in motor deficits. Guo-mediated prevention of apoptotic cell death in ischemic area. Maintenance of BBB integrity after ischemic insult in Guo treated animals. Quantitative EEG study results: partial prevention of global, bilateral state of synchronicity (hyperexcitability) induced by ischemic in Guo treated animals.	Müller et al. (2020) [163]

Abbreviations: Guo—Guanosine; I.p.—Intraperitoneal; I.n.—Intranasal; PT—Photothrombotic Stroke; MCAo—Middle Cerebral Artery Occlusion; ROS—Reactive Oxygen Species; SOD—Superoxide Dismutase; CAT—Catalase; GLT -1—Glutamate Transporter 1; GS—Glutamine Synthetase; VEGF—Vascular Endothelial Growth Factor; BNDF—Brain Derived Neurotrophic Factor; BBB—Blood–Brain Barrier; EEG—Electroencephalography.

## Data Availability

Not applicable.
